# Bayesian Multi-Model Comparison and Nonlinear Mixed Modelling of Growth Trajectories in Denizli Chickens

**DOI:** 10.3390/ani16111633

**Published:** 2026-05-27

**Authors:** Harun Raşit Manav, Doğan Narinç, Ali Aygun, Nihan Öksüz Narinç, Ebru Kaya Başar, Mehmet Ziya Fırat

**Affiliations:** 1 Aselya Agriculture Corporation, Antalya 07500, Turkey; hrmanav@hotmail.com; 2 Department of Animal Science, Faculty of Agriculture, Akdeniz University, Antalya 07070, Turkey; mzfirat@akdeniz.edu.tr; 3 Department of Animal Science, Faculty of Agriculture, Selçuk University, Konya 42130, Turkey; aaygun@selcuk.edu.tr; 4 Department of Finance and Banking, Faculty of Applied Sciences, Akdeniz University, Antalya 07070, Turkey; nihanoksuz@akdeniz.edu.tr; 5 Statistical Consulting Application and Research Center, Akdeniz University, Antalya 07070, Turkey; ebrukaya@akdeniz.edu.tr

**Keywords:** Bayesian modelling, indigenous chicken, growth trajectory, production system, nonlinear mixed model, sexual dimorphism

## Abstract

This study aimed to model the growth trajectories of Denizli chickens under different production systems and to identify the most appropriate nonlinear growth function within a Bayesian framework. A total of 156 birds were monitored weekly from hatch to 26 weeks of age under conventional cage, conventional floor, and enriched floor systems. Eight candidate nonlinear growth models were evaluated using Bayesian model comparison criteria, including leave-one-out cross-validation (LOO) and the widely applicable information criterion (WAIC). Among the evaluated models, the Gompertz function showed the best predictive performance, with the lowest LOOIC (225.16) and superior predictive accuracy across fit statistics. The selected model was subsequently extended to a Bayesian nonlinear mixed modelling framework to evaluate the effects of sex and production system on growth dynamics while accounting for between-animal variability. Males exhibited substantially higher asymptotic weights than females, whereas females showed faster early growth and earlier stabilization. Birds reared under the conventional floor system, particularly males, exhibited the highest asymptotic growth potential and later inflection ages, indicating a more prolonged growth phase. In contrast, enriched systems appeared to have promoted greater variability in growth responses, possibly due to increased behavioral activity and energy expenditure. The findings demonstrated that production system and sex jointly influenced both the scale and timing of growth in Denizli chickens. Beyond statistical model comparison, the Bayesian nonlinear mixed modelling approach provided biologically meaningful information that could support breeding, housing, and management decisions for indigenous and dual-purpose poultry production systems.

## Simple Summary

This study showed that growth in Denizli chickens differed according to sex and housing system. Males grew larger but more slowly, whereas females matured earlier. Bayesian modelling revealed that growth rate and growth timing jointly shaped developmental patterns under different production conditions.

## 1. Introduction

Poultry production systems are undergoing a significant transformation driven by the increasing demand for sustainable and resilient animal production strategies. In this context, the utilization of dual-purpose genotypes has gained renewed attention as an alternative to highly specialized commercial hybrids, particularly in response to concerns regarding sustainability, animal welfare, and resource efficiency. Dual-purpose chickens provide a balanced potential for both meat and egg production while contributing to genetic diversity and production system resilience. Moreover, the conservation and effective utilization of local genetic resources have been widely recognized as essential components of sustainable livestock production, as these genotypes often exhibit enhanced adaptability to local environmental conditions and management practices compared to highly selected commercial lines [1,2,3].

Among local genetic resources, indigenous chicken breeds represent a valuable yet underutilized component of sustainable poultry production systems. These genotypes are typically characterized by their adaptability to diverse environmental conditions, disease resistance, and ability to perform under low-input management systems, making them particularly relevant for alternative and resilient production strategies [2,3]. The Denizli chicken, one of the most well-known indigenous breeds in Türkiye, has traditionally been recognized for its unique phenotypic traits and cultural importance [4,5]. Despite its potential as a dual-purpose genotype, the production performance of Denizli chickens remains relatively underexplored compared to commercial hybrids. In particular, studies focusing on growth dynamics and quantitative modelling of body weight development in this breed are scarce, limiting the ability to fully evaluate its production potential and to design effective breeding and management strategies. More importantly, existing studies on poultry growth have predominantly focused on commercial breeds and have typically relied on single-model approaches, without systematically evaluating alternative growth functions within a probabilistic framework. As a result, there is limited understanding of how model uncertainty may influence biological interpretation, particularly in local chicken breeds raised under different production systems.

Growth performance in poultry is not solely determined by genetic background but is also strongly influenced by environmental and management-related factors. In poultry science, the term “production system” generally refers to the combination of housing structure, environmental management, stocking density, and husbandry practices under which birds are reared. Differences among production systems may substantially influence bird behavior, activity level, welfare status, feed utilization, and growth performance by modifying both the physical and social environment experienced by the animals [6,7]. Among these, the housing system has been identified as a key determinant affecting growth rate, feed efficiency, and overall production performance. Differences between conventional cage systems and alternative rearing systems, such as deep litter or enriched environments, may lead to substantial variation in growth trajectories due to differences in activity levels, space allowance, and micro-environmental conditions [6,7]. Such environmental heterogeneity can result in distinct growth patterns even within the same genotype, highlighting the importance of appropriately modelling growth responses under varying production conditions.

Mathematical modelling of growth trajectories has long been an essential tool in poultry science for describing and predicting body weight development over time. Nonlinear growth models such as Gompertz, Logistic, and von Bertalanffy functions have been widely applied due to their biological interpretability and ability to capture sigmoidal growth patterns [8,9]. Among these, the Gompertz model has been particularly favored in poultry studies because of its flexibility and relatively good fit to empirical data [8,10]. However, despite their widespread use, these classical models often impose restrictive assumptions, such as fixed inflection points or limited flexibility in curve shape, which may not adequately represent the full variability observed in growth trajectories across different genotypes and production conditions. Consequently, reliance on a single predefined model may lead to biased inference and suboptimal representation of biological growth processes.

In recent years, Bayesian approaches have emerged as powerful alternatives for modelling biological growth processes, offering a flexible framework that allows for the incorporation of prior information and a full probabilistic description of parameter uncertainty. Unlike traditional frequentist methods, Bayesian inference enables the estimation of posterior distributions for model parameters, thereby providing more informative insights into growth dynamics and prediction uncertainty [11,12]. In addition, the use of model comparison criteria such as the widely applicable information criterion (WAIC) [13] and leave-one-out cross-validation (LOO) [14] allows for a more robust evaluation of competing nonlinear models beyond conventional goodness-of-fit measures. Importantly, selecting the most appropriate functional form is a critical prerequisite for subsequent modelling stages, particularly when extending analyses to nonlinear mixed modelling frameworks that incorporate biological sources of variation such as sex and production system effects. Previous studies have demonstrated that nonlinear mixed models substantially improve model accuracy by accounting for between-individual variability and heterogeneity in growth data [15]. Furthermore, the integration of biological covariates such as sex within nonlinear mixed modelling frameworks has been shown to provide more accurate and biologically meaningful interpretations of growth dynamics [16]. More recently, Bayesian approaches have been successfully applied for nonlinear model selection and inference in poultry growth studies, offering enhanced flexibility and improved uncertainty quantification [17]. In this context, integrating Bayesian multi-model comparison with subsequent nonlinear mixed modelling provides a comprehensive and coherent strategy, enabling both optimal model selection and biologically meaningful inference while accounting for parameter and model uncertainty.

Despite these advances, there remains a lack of integrated frameworks that simultaneously address model uncertainty and biological variability in poultry growth, particularly in local breeds under alternative production systems. This limitation restricts the ability to draw robust and generalizable conclusions about growth dynamics.

It was hypothesized that growth trajectories in Denizli chickens would differ according to sex and production system, and that Bayesian nonlinear mixed modelling would provide a biologically meaningful and statistically robust characterization of growth dynamics by simultaneously accounting for model uncertainty and between-animal variability. The aim of this study was to model the growth trajectory of Denizli chickens using a Bayesian framework and to identify the most appropriate nonlinear growth function among multiple candidate models. For this purpose, eight commonly used nonlinear growth models were fitted and compared using Bayesian model evaluation criteria, including WAIC and LOO. In a subsequent stage, the selected best-fitting model was extended to a nonlinear mixed modelling framework to investigate the effects of biological and environmental factors, specifically sex and rearing system, on growth dynamics. This two-stage approach not only enabled robust selection of the most appropriate growth function but also provided deeper biological insight into how growth scale and timing were jointly regulated under different environmental conditions. By explicitly integrating model uncertainty and individual-level variability, this framework offered a more comprehensive understanding of growth dynamics compared to traditional approaches.

## 2. Materials and Methods

### 2.1. Experimental Animals and Management

A total of 156 purebred Denizli chickens obtained from hatching eggs sourced from the Denizli Provincial Directorate of Agriculture and Forestry breeding flock were used as animal material in this study. All procedures were approved by the Animal Experiments Local Ethics Committee (Approval No: B.30.2.AKD.0.05.07.00/50). Additionally, the study was carried out under the authorization of the Ministry of Agriculture and Forestry (Permission No: E-22875267-325.04.02-3661514). All experiments were performed in accordance with the relevant guidelines and regulations.

The study was conducted at the Poultry Research Center of Akdeniz University, Turkey. At hatch, all chicks were individually wing-banded to enable longitudinal tracking and pedigree recording. During the brooding period (first three weeks), chicks were housed in environmentally controlled multi-tier battery cages with compartment dimensions of 96 × 43 × 21 cm. Each compartment contained 10 chicks, corresponding to a stocking density of approximately 475 cm^2^ per bird. Ambient temperature was maintained at 32 °C during the first five days and gradually reduced by 1 °C every four days, reaching 27 °C at the end of the third week. Following the brooding phase, birds were randomly allocated to three different production systems: conventional deep litter, enriched deep litter, and conventional multi-tier cage system. Sex determination was performed during the early growth period based on secondary sexual characteristics, including comb and wattle development, plumage characteristics, and body size differences. Both sexes were represented across all production systems and experimental units. In both deep litter systems, birds were housed in floor pens with a surface area of 3 m^2^ at a density of 7 birds per m^2^. Random allocation was performed to ensure balanced distribution of birds across production systems and experimental units while minimizing potential allocation bias. Bedding material consisted of wood shavings with a depth of approximately 4 cm. The enriched deep litter system included additional environmental enrichments such as perches, dust-bathing areas, abrasive devices for beak and claw conditioning, and suspended objects to stimulate exploratory behavior.

The cage system consisted of three-tier cage units organized into four physically separated cage compartments, housing a total of 72 birds under stocking density conditions comparable to those of the floor systems. All experimental units were equipped with automatic feeding and watering systems and operated under controlled environmental conditions, ensuring comparable climatic conditions across treatments. All housing systems were maintained within the same environmentally controlled research facility under standardized ventilation and lighting management conditions. Environmental conditions, including litter quality, ventilation adequacy, and general air quality indicators, were monitored daily throughout the experimental period to ensure comparable microenvironmental conditions among treatments. Because the housing structure did not provide fully independent replicated block units across all production systems, a formal randomized complete block design was not implemented. Instead, birds were randomly allocated under standardized environmental conditions, and the hierarchical longitudinal structure of the data was addressed using Bayesian nonlinear mixed modelling with individual-level random effects. The same photoperiod and artificial lighting program were applied across all production systems. A lighting program of 23 h light per day was applied during the first three days, after which the photo period was gradually reduced to 18 h/day by day 10 and was maintained thereafter. Feed and water were provided ad libitum throughout the experiment. Birds were fed three-phase diets: starter diet (0–3 weeks; 21% crude protein, 3000 kcal ME/kg), grower diet (4–12 weeks; 20% crude protein, 2900 kcal ME/kg), and developer diet (12–26 weeks; 18% crude protein, 2800 kcal ME/kg). Dietary transitions between feeding phases were implemented gradually, and no marked growth depression or growth discontinuity associated with dietary phase changes was observed during the experimental period.

### 2.2. Data Collection and Preparation

Individual body weights were recorded weekly from hatch (week 0) to 26 weeks of age, resulting in longitudinal growth trajectories for each bird. The dataset included repeated measurements across time, nested within individuals, and structured according to sex and production system.

Prior to analysis, the dataset was reorganized from wide to long format, where each row corresponded to a single observation (bird × week). The final dataset contained the following variables: individual identifier, sex, production system, week of age, and body weight. Because each bird was measured repeatedly from hatch to 26 weeks of age, the dataset contained longitudinal repeated observations that provided substantial inferential information for estimating growth trajectories and interaction effects within the Bayesian nonlinear mixed modelling framework.

Data quality control procedures included removal of missing values, verification of measurement consistency, and alignment of time indices across individuals. No artificial smoothing or transformation was applied to preserve the biological variability inherent in growth processes.

### 2.3. Bayesian Nonlinear Model Comparison

To identify the most appropriate functional form for describing growth trajectories, a set of candidate nonlinear growth models was evaluated within a Bayesian framework. The models included commonly used sigmoidal growth functions with both fixed and flexible inflection points, namely Gompertz, Logistic, von Bertalanffy, Richards, Weibull, Brody, Negative Exponential, and Morgan–Mercer–Flodin (MMF) models. The mathematical forms of the evaluated models are given below, where $W_{t}$ represents body weight at time t:
Gompertz model:$W_{t}=A\cdot exp\left(-B\cdot exp\;(-kt)\right)$Logistic model:$W_{t}=\frac{A}{1+B\cdot exp\;(-kt)}$von Bertalanffy model:$W_{t}=A\cdot {\left(1-B\cdot exp\;(-kt)\right)}^{3}$Richards model:$W_{t}=A\cdot {\left(1+B\cdot exp\;(-kt)\right)}^{-1/m}$Weibull model:$W_{t}=A\cdot \left(1-exp\;(-kt^{m})\right)$Brody model:$W_{t}=A\cdot \left(1-B\cdot exp\;(-kt)\right)$Negative exponential model:$W_{t}=A\cdot \left(1-exp\;(-kt)\right)$Morgan–Mercer–Flodin (MMF) model:$W_{t}=\frac{A\cdot B+k\cdot t^{m}}{B+t^{m}}$

In these models, A denotes the asymptotic body weight, representing the model-based upper growth limit approached as age increases. The parameter k is a growth-rate-related parameter that controls the speed at which body weight approaches the asymptote; however, its exact interpretation depends on the mathematical form of the model. The parameter B is an integration or shape-related constant affecting the initial position and horizontal displacement of the growth curve, whereas m, when present, is an additional shape parameter controlling curve flexibility and inflection characteristics. For the Morgan–Mercer–Flodin (MMF) model, the parameters k and B should not be interpreted directly as biological growth-rate parameters analogous to those used in Gompertz or Logistic models. Instead, these parameters jointly influence the scaling, curvature, and temporal transition characteristics of the growth trajectory within the MMF functional form. For all candidate growth models, the observed body weight at time t for individual i was modelled as:

$$W_{it}=f(t,\theta_{i})+\varepsilon_{it}$$

where $f(t,\theta_{i})$ represents the corresponding nonlinear growth function, and residual errors were assumed to follow a Gaussian distribution:

$$\varepsilon_{it}∼N(0,\sigma^{2})$$

with $\sigma^{2}$ estimated within the Bayesian framework. Residual errors were modelled under a homoscedastic Gaussian assumption, with a constant residual variance ($\sigma^{2}$) across the longitudinal observation period.

Each model was fitted within a Bayesian framework using the brms package in R, which interfaces with Stan for posterior sampling via Hamiltonian Monte Carlo (HMC) and the No-U-Turn Sampler (NUTS) [18,19,20,21]. Weakly informative priors were specified on the log scale to ensure biologically realistic estimates while avoiding over-regularization [11,22]. For log(A), the intercept prior was Normal (8.330, 0.5), whereas fixed-effect coefficients were assigned Normal (0, 0.35) priors. For log(k), the intercept prior was Normal (−1.897, 0.6), with Normal (0, 0.35) priors for fixed-effect coefficients. For log(B), the intercept prior was Normal (1.253, 0.7), and fixed-effect coefficients were assigned Normal (0, 0.4) priors. Standard deviations of individual-level random effects for log(A) and log(k) were assigned half Student-*t* priors, Student-*t* (3, 0, 0.5), whereas the residual standard deviation was assigned Student-*t* (3, 0, 150). Logarithmic parameterization was preferred because growth parameters are inherently positive, and modelling them on the log scale improves numerical stability while preventing biologically implausible parameter exploration during posterior sampling. Prior means were centered around biologically plausible values for Denizli chicken growth trajectories, while prior variances were intentionally kept broad to maintain weak regularization. Preliminary sensitivity evaluations using alternative prior variances yielded highly consistent posterior estimates, indicating that the main inferences were robust to moderate changes in prior specification. For each model, four Markov Chain Monte Carlo (MCMC) chains were run with 6000 iterations per chain, including a warm-up (burn-in) period of 3000 iterations, resulting in 12,000 posterior samples for inference. Convergence was considered satisfactory when R^ < 1.01 for all parameters, bulk ESS > 400, and tail ESS > 400 [23]. No divergent transitions were observed across any model, indicating adequate exploration of the posterior geometry. All reported 95% credible intervals (CrI) were calculated as quantile-based equal-tailed intervals derived from the 2.5th and 97.5th percentiles of the posterior distributions.

Model comparison was based primarily on approximate leave-one-out cross-validation (LOO) using expected log predictive density (ELPD), while the widely applicable information criterion (WAIC) was also reported as a complementary Bayesian information criterion [13,14]. In addition to LOO, model performance was evaluated using multiple goodness-of-fit and predictive accuracy criteria, including root mean square error (RMSE), mean absolute error (MAE), and Bayesian $R^{2}$. These complementary metrics were used to assess both in-sample fit and out-of-sample predictive performance, providing a comprehensive evaluation of competing growth models.

The best-fitting model was selected based on a combination of LOO-based criteria and predictive accuracy measures, prioritising models that achieved superior predictive performance while maintaining biological interpretability and parameter stability.

### 2.4. Selection of Best Model and Nonlinear Mixed Modelling

Following Bayesian model comparison, the most appropriate functional form was selected based on predictive performance and model stability criteria. This selected model was subsequently used as the basis for nonlinear mixed modelling.

To account for the longitudinal and hierarchical structure of the data, a Bayesian nonlinear mixed model (NLMM) framework was implemented. In this framework, key growth parameters were modelled as functions of sex, production system, and their interaction, while individual-level random effects were included to capture between-animal variability in growth trajectories.

Specifically, asymptotic body weight and growth rate parameters were modelled with both fixed and random effects, whereas the integration constant was modelled using fixed effects only. This modelling choice was made to balance biological interpretability with computational efficiency and parameter identifiability. This specification was based on the biological relevance of these parameters, as asymptotic weight and growth rate are known to exhibit substantial between-individual variability, whereas the integration constant is typically less variable and more difficult to estimate reliably as a random parameter. Because inflection-related traits in the Gompertz model are jointly determined by both B and k, modelling individual-level variability in k already captures an important component of between-animal variation in inflection timing. Therefore, retaining B as a fixed-effect parameter was considered sufficient to preserve biologically meaningful variability while maintaining parameter identifiability and computational stability. Alternative model formulations, including an individual-level random effect for log(B), were also evaluated during model development. However, these formulations did not meaningfully improve model adequacy or predictive performance and resulted in weaker parameter identifiability and less stable posterior behavior. Therefore, the final model retained individual-level random effects for log(A) and log(k), while log(B) was modelled using fixed effects only.

Logarithmic transformations were applied to all nonlinear parameters to ensure positivity and improve numerical stability. The final model structure was defined as:

log(A) ~ sex × production system + (1 | individual);

log(k) ~ sex × production system + (1 | individual);

log(B) ~ sex + production system.

Bayesian inference was performed using Hamiltonian Monte Carlo sampling via the brms package with the CmdStanR backend [24,25].

Four Markov chains were run with 6000 iterations per chain, including 3000 warm-up iterations, yielding 12,000 posterior samples.

Model convergence and sampling adequacy were assessed using the potential scale reduction factor (R^), effective sample size (ESS), and inspection of sampling diagnostics. Model fit was evaluated using posterior predictive checks by comparing observed and simulated data distributions, including graphical assessment of predicted versus observed trajectories and residual patterns. In addition, Bayesian R^2^ was used to quantify the proportion of variance explained by the model.

Derived biological parameters, including asymptotic weight, growth rate, and inflection point characteristics, were calculated from posterior samples for subsequent interpretation. Inflection age was defined as the age at which the instantaneous growth rate reached its maximum, corresponding to the point where the second derivative of the fitted growth function equals zero. Inflection weight was defined as the predicted body weight at this age. For the final Gompertz model, inflection age and inflection weight were calculated as $t_{i}=ln(B)/k$ and $W_{i}=A/e$, respectively. Inflection-related traits were calculated from posterior samples, allowing uncertainty in these derived parameters to be propagated into their credible intervals. For non-sigmoidal models without a finite inflection point, such as Brody and negative exponential functions, inflection traits were not interpreted biologically. All estimated inflection ages obtained from the final Gompertz nonlinear mixed model remained within the observed experimental period (0–26 weeks), indicating that inflection-related traits were estimated from data-supported regions of the growth trajectory rather than from extreme extrapolation.

## 3. Results

### 3.1. Model Comparison and Fit Diagnostics

Eight nonlinear growth models were evaluated within a Bayesian framework using multiple model selection and predictive performance criteria. Model comparison based on approximate leave-one-out cross-validation (LOO) clearly indicated that the Gompertz model provided the best fit to the data, with the lowest LOOIC value (225.16) and highest predictive accuracy (Table 1).

The superiority of the Gompertz model was consistent across all evaluation metrics. Compared to the second-best model (Richards), the Gompertz model showed a ΔLOOIC of 10.91, indicating a substantial improvement in predictive performance. Larger differences were observed for all remaining models, with ΔLOOIC values exceeding 30 for MMF and Weibull, and exceeding 90 for Negative Exponential and Brody models.

Predictive accuracy metrics further supported these findings. The Gompertz model achieved the lowest RMSE (13.06) and MAE (8.95), indicating superior agreement between observed and predicted values. In addition, the model exhibited the highest Bayesian R^2^ (0.9997), reflecting an excellent ability to capture the variability in body weight data.

All models demonstrated satisfactory convergence diagnostics, with R^ values close to 1.00, high effective sample sizes, and no divergent transitions observed, indicating stable posterior sampling and reliable parameter estimation. These results provided strong and consistent evidence that the Gompertz function is the most appropriate model for describing growth trajectories in Denizli chickens, and it was therefore selected for subsequent nonlinear mixed modelling.

### 3.2. Population-Level Parameter Estimates

Following model selection, the Gompertz model was extended to a Bayesian nonlinear mixed-effects framework, and population-level parameter estimates were obtained (Table 2). At the baseline level, the posterior mean of the asymptotic parameter (log A) was estimated as 7.867 (95% CrI: 7.856–7.879), corresponding to an asymptotic body weight of approximately 2600 g. The growth rate parameter (log k) had a posterior mean of −1.954 (95% CrI: −1.980 to −1.930), indicating a moderate growth velocity. The integration constant (log B) was estimated at 1.239 (95% CrI: 1.216–1.263), reflecting the curvature and timing of the growth trajectory. The residual standard deviation (σ) was estimated at 14.64 (95% CrI: 11.03–19.92), indicating a relatively low level of unexplained variability around the fitted growth curves. The narrow credible intervals and low posterior uncertainty associated with all parameters suggest that the model provided precise and stable estimates of growth dynamics at the population level.

### 3.3. Group-Specific Growth Dynamics

Substantial differences in growth dynamics were observed across production systems and sex (Table 3). Across all production systems, males consistently exhibited higher asymptotic weights (A) than females. The highest asymptotic weight was observed in males reared under the conventional floor system (3322 g; 95% CrI: 3077–3576), whereas the lowest values were recorded in females housed in the conventional cage system (2159 g; 95% CrI: 2048–2278). Growth rate (k) showed an inverse relationship with asymptotic weight. Females generally exhibited higher growth rates than males across systems, with the highest k value observed in females under cage conditions (0.150; 95% CrI: 0.146–0.153). In contrast, males reared on the floor system displayed the lowest growth rate (0.129; 95% CrI: 0.125–0.133). Inflection age followed a similar pattern, with males reaching the inflection point later than females across all systems. The latest inflection was observed in males under the floor system (9.75 weeks; 95% CrI: 9.43–10.08), whereas females reached the inflection point earlier, particularly under cage conditions. Inflection weight was consistently higher in males, reflecting their greater growth potential. The highest value was observed in males reared on the floor system (1222 g; 95% CrI: 1132–1316), while females showed lower and relatively stable values across systems.

### 3.4. Model Fit and Growth Curve Evaluation

The population-level fitted Gompertz curves showed close agreement with the observed mean body weight trajectories across sex and production-system combinations. Overall, 88.9% of the observed group means fell within the 95% credible intervals of the fitted curves, indicating satisfactory calibration of the nonlinear mixed model.

**Figure 1:**
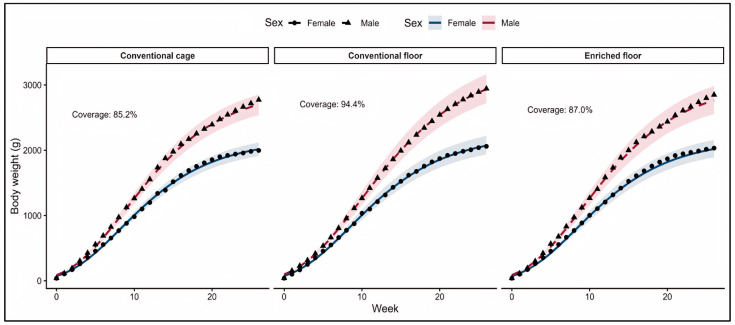
Observed and fitted growth trajectories of Denizli chickens across production systems and sex. Lines represented posterior mean predictions from the population-level Gompertz nonlinear mixed-effects model, shaded bands indicated 95% credible intervals, and points represented observed group mean body weights. Coverage values indicated the proportion of observed means falling within the 95% credible intervals.

Model fit was strongest for birds reared under the conventional floor system, particularly in males, where the agreement between observed and fitted values was highest across weeks (Figure 1). In contrast, somewhat larger deviations were observed in the enriched floor groups, especially in males, although the overall sigmoidal growth pattern remained well captured.

Across all systems, the fitted curves successfully reproduced the biologically expected separation between sexes. Male trajectories remained above female trajectories throughout most of the growth period and exhibited a more prolonged growth phase, whereas females showed steeper early growth and earlier stabilization. Group divergence became particularly evident during the intermediate growth phase, consistent with the posterior estimates of growth rate and inflection age.

**Figure 2:**
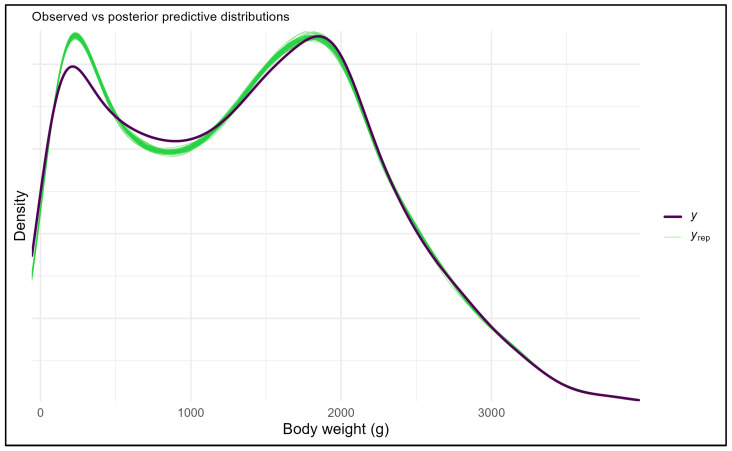
Posterior predictive check comparing the observed body weight distribution (dark line) with replicated data simulated from the fitted Bayesian Gompertz model (green lines). The close overlap between observed and predicted distributions indicated that the model adequately captures the overall distributional structure of the data.

The adequacy of the model was further evaluated using posterior predictive checks. The posterior predictive distributions closely matched the observed data distribution, indicating that the model successfully captured not only the mean growth trajectory but also the overall distributional characteristics of body weight (Figure 2). Minor deviations were observed in the lower weight range, although these differences were small and did not affect the overall model performance.

**Figure 3:**
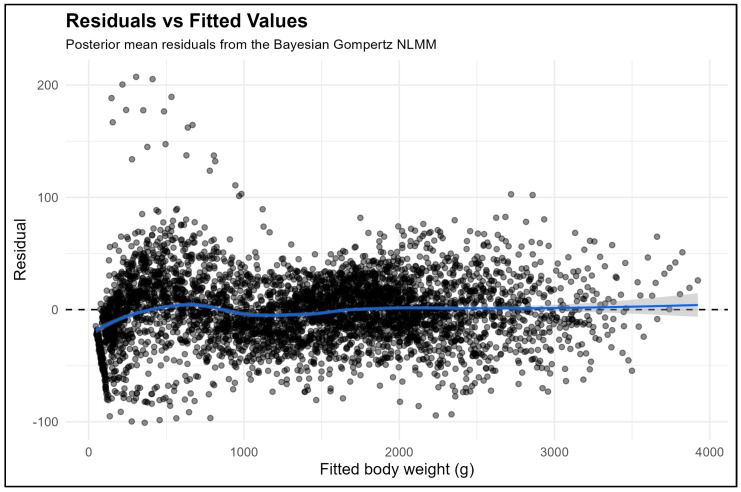
Residuals versus fitted values from the Bayesian Gompertz nonlinear mixed-effects model. The absence of a systematic pattern and the near-zero smoothed trend indicated that the model adequately captures the underlying structure of the data, with no evidence of major heteroscedasticity or model misspecification.

In addition to posterior predictive checks, residual diagnostics were examined to further assess model adequacy. The residuals showed no clear systematic pattern across fitted values, and the smoothed trend remained close to zero throughout the range of predicted body weights (Figure 3). Although slightly higher variability was observed at lower weight ranges, this pattern is expected in biological growth data and did not indicate model misspecification. Overall, the residual structure supported the assumption of an adequate model fit and suggested that the Gompertz nonlinear mixed model successfully captured the main sources of variation in the data.

### 3.5. Posterior Contrasts and Statistical Evidence

Posterior contrast analysis provided strong statistical support for sex-related differences within each production system (Table 4). For asymptotic body weight (A), males consistently exceeded females in all systems, with posterior mean differences of 780.23 g in the conventional cage system, 1080.49 g in the conventional floor system, and 868.18 g in the enriched floor system. In all cases, the 95% credible intervals excluded zero and the posterior probability that the male–female difference was positive was 1.00.

A similar pattern was observed for inflection-related traits. Males reached the inflection point later than females by 0.61 weeks in the conventional cage system, 1.28 weeks in the conventional floor system, and 0.74 weeks in the enriched floor system. Likewise, inflection weight was markedly higher in males, with posterior mean differences of 287.03 g, 397.49 g, and 319.39 g in the conventional cage, conventional floor, and enriched floor systems, respectively. All of these contrasts were also strongly supported by 95% credible intervals excluding zero and posterior probabilities equal to 1.00.

In contrast, the growth rate parameter (k) was consistently lower in males than in females. Male–female differences in k were estimated as −0.0068 in the conventional cage system, −0.0158 in the conventional floor system, and −0.0086 in the enriched floor system, with all 95% credible intervals remaining below zero. Accordingly, the posterior probabilities that these male–female differences were greater than zero were close to zero, indicating strong posterior support for faster early growth in females despite their lower asymptotic body weight.

In addition to the primary growth parameters, the integration constant (B) was consistently higher in males across all production systems, with 95% credible intervals excluding zero and posterior probabilities equal to 1.00 (Table 4). Although B does not have a direct biological interpretation, it played an important role in determining the horizontal positioning of the growth curve. In the present study, the higher B values observed in males contributed to a rightward shift in the growth trajectory, which was consistent with the delayed inflection age estimated for males. This result indicated that differences in B, together with growth rate (k), jointly shaped the temporal dynamics of growth rather than representing an independent biological effect.

## 4. Discussion

The observed dominance of the Gompertz model was highly consistent with previous literature, where it has repeatedly been identified as one of the most suitable models for describing poultry growth due to its biological interpretability and ability to capture sigmoidal growth dynamics. For example, Galeano-Vasco et al. [26] reported that the Gompertz model outperformed alternative nonlinear functions, such as Logistic and Brody, in laying hens based on information criteria. Similarly, Santos et al. [10] showed that the Gompertz model provided the best fit for quail growth under a mixed-model framework, highlighting its capacity to represent variability across genetic backgrounds. From a Bayesian perspective, Gotuzzo et al. [27] also showed that the Gompertz model achieved superior model adequacy, as indicated by lower deviance-based criteria across different growth phases. Taken together, these findings reinforced the robustness of the Gompertz function across species, production conditions, and statistical paradigms.

Although more flexible models such as Richards or Weibull may provide improved fit in certain datasets, particularly when growth trajectories deviate from standard sigmoid shapes [28], their advantages are often offset by increased model complexity and potential instability in parameter estimation. In particular, the Richards model, despite its flexibility due to the additional shape parameter, is often associated with convergence difficulties and parameter identifiability issues, especially in Bayesian frameworks where posterior distributions may become poorly defined. In contrast, the Gompertz model offered a parsimonious structure with biologically interpretable parameters, which became especially advantageous when integrated into hierarchical Bayesian frameworks. Moreover, its suitability for describing single-phase growth processes, as commonly observed in poultry raised under non-reproductive conditions, further supported its selection in the present study [29]. Taken together, the results confirmed that the Gompertz function provided a robust, interpretable, and computationally stable foundation for modeling poultry growth within a Bayesian nonlinear mixed modeling context. Although the Richards model showed relatively competitive predictive performance, the substantially lower LOOIC values, superior predictive accuracy metrics, and greater computational stability observed for the Gompertz model supported its selection as the primary modelling framework. In addition, the more parsimonious structure and stronger biological interpretability of the Gompertz function made it particularly suitable for subsequent nonlinear mixed modelling applications. The MMF model exhibited greater parameter dependency and comparatively lower sampling efficiency than simpler sigmoidal models, particularly due to posterior correlations among shape-related parameters. This behavior was consistent with the known identifiability challenges associated with highly flexible nonlinear growth functions and likely contributed to the lower computational stability of the MMF model. Despite their increased flexibility, the Richards and MMF models provided only limited improvement in predictive fit relative to their higher effective model complexity penalized by LOOIC and WAIC. This finding suggested that the additional flexibility of four-parameter growth functions did not substantially improve out-of-sample predictive performance for the present data.

A key strength of the present study lies in the integration of Bayesian inference with nonlinear mixed modeling, which enabled a comprehensive characterization of growth dynamics beyond traditional fixed-effect approaches. Previous studies have demonstrated that mixed-model formulations of growth functions can substantially reduce residual variance and improve goodness-of-fit by capturing individual heterogeneity in growth trajectories [30,31]. Similarly, Camargo Júnior et al. [32] emphasized that assigning random effects to biologically relevant parameters such as asymptotic weight and growth rate enhances model flexibility and predictive performance. The present findings were fully consistent with these reports, as the inclusion of individual-level random effects in the Gompertz model resulted in stable convergence, high effective sample sizes, and accurate representation of observed growth patterns.

The Bayesian framework further strengthened the analytical approach by enabling full probabilistic inference through posterior distributions. Unlike classical methods, Bayesian models allowed direct quantification of uncertainty and facilitated formal evaluation of parameter differences using credible intervals and posterior probabilities. In addition, the use of predictive criteria such as LOO and WAIC provided a robust basis for model comparison, combining statistical rigor with biological interpretability. Importantly, the two-stage framework implemented in this study—first identifying the most appropriate growth function and subsequently extending it within a nonlinear mixed-effects structure—offered a coherent and methodologically rigorous approach that addresses both model uncertainty and biological variability simultaneously, which has been rarely achieved in previous poultry growth studies.

From a biological perspective, the results revealed a clear and consistent pattern of sexual dimorphism in growth dynamics. Males exhibited substantially higher asymptotic weights than females across all production systems, with differences ranging from 780 g in cage systems to over 1080 g under conventional floor conditions. These magnitudes indicated a biologically meaningful divergence rather than a marginal effect. In contrast, females consistently exhibited higher growth rate parameters (k), indicating faster early development but earlier stabilization. This inverse relationship between growth rate and asymptotic size was consistent with previous reports in poultry and other livestock species, where rapid early growth is often associated with reduced final body weight [10]. The combination of high growth rate but low asymptotic weight observed in cage-reared females suggests accelerated early maturation accompanied by earlier stabilization of growth. This pattern may have reflected a physiological trade-off in which rapid early growth is achieved at the expense of prolonged body mass accumulation, resulting in lower final growth potential. A biologically consistent association was also observed between asymptotic weight and inflection-related traits. Groups characterized by higher asymptotic weights generally exhibited later inflection ages and higher inflection weights, indicating a more prolonged growth phase before reaching maximal growth velocity. This pattern suggested that birds with greater mature body size maintain active body mass accumulation for a longer duration, which was consistent with classical poultry growth dynamics.

Importantly, the present study extended this interpretation by demonstrating that sexual dimorphism cannot be explained solely by differences in asymptotic weight. The integration constant (B), which is frequently overlooked in poultry growth studies, was consistently higher in males across all production systems, with credible intervals excluding zero and posterior probabilities equal to 1.00. Although B does not have a direct, standalone biological interpretation, it played a critical role in determining the temporal positioning of the growth curve. When interpreted jointly with k, the observed pattern of higher B and lower k in males indicates a systematic rightward shift in the growth trajectory, corresponding to delayed inflection age and prolonged growth duration. This finding suggested that differences in B and k jointly regulate the temporal organization of growth trajectories, rather than representing independent effects. Such a joint interpretation of growth parameters remained underexplored in poultry literature and constitutes one of the key contributions of the present study.

Production system effects also contributed to variation in growth dynamics, although their magnitude was smaller than that of sex. The highest asymptotic weights were observed in birds reared under the conventional floor system, particularly in males (3322 g), compared to cage (2940 g) and enriched systems (3041 g). These differences were substantial and indicated that housing conditions can meaningfully alter growth potential rather than merely introducing minor variation. A similar pattern was observed for inflection weight, further supporting the conclusion that the floor system promoted a more prolonged and sustained growth phase. Although male birds in the floor system still exhibited slight increases in body weight toward the end of the experimental period, the growth trajectories showed substantial deceleration during the final weeks, indicating progressive convergence toward the asymptotic phase. Therefore, the estimated asymptotic weights should be interpreted as model-based theoretical growth limits rather than strictly observed terminal body weights.

The biological mechanisms underlying these differences were likely related to variation in activity levels, energy expenditure, and resource utilization across production systems. Floor-based systems may have allowed more natural movement and behavioral expression, potentially improving muscle development and growth efficiency, whereas enriched systems may introduce greater variability due to increased environmental complexity. The slightly lower model fit observed in enriched groups, particularly in males, supported this interpretation and suggested increased heterogeneity in growth responses under these conditions. Similar inconsistencies in housing effects have been reported in previous studies [6], highlighting the complex interaction between environment, behavior, and physiology in poultry production. One possible explanation for the slightly lower asymptotic weights observed in enriched males is the increased locomotor and exploratory activity associated with environmental enrichment. Greater behavioral activity may increase maintenance energy expenditure and reduce the proportion of available energy allocated to long-term body mass accumulation.

Another important implication of the present findings is that growth differences among groups become more pronounced during the intermediate growth phase rather than at early stages. This observation underscored the importance of modeling the entire growth trajectory rather than relying on single time-point comparisons. Growth curve approaches provided a more comprehensive understanding of developmental dynamics by capturing both the scale and timing of growth, which are jointly determined by multiple parameters. Compared with highly selected commercial broiler genotypes, Denizli chickens exhibited relatively slower and more prolonged growth trajectories, which is consistent with the expected biological characteristics of indigenous dual-purpose breeds. The delayed inflection ages and moderate asymptotic growth observed in the present study are also generally coherent with previous reports on native Turkish chicken populations, reflecting lower selection intensity for rapid juvenile growth and greater adaptation to alternative production systems. In contrast to commercial dual-purpose hybrids, Denizli chickens appeared to maintain a more extended growth phase with reduced early growth intensity, particularly in males. From a practical perspective, the present findings suggested that conventional floor systems may provide the most suitable housing strategy for small-scale producers aiming to maximize growth performance and final body weight in Denizli chickens, particularly in males. In contrast, enriched systems may have offered welfare-related advantages through increased behavioral activity and environmental complexity, although this may be accompanied by slightly reduced growth efficiency due to higher maintenance energy expenditure.

## 5. Conclusions

The present study demonstrated that the Gompertz model provided the most reliable representation of growth in Denizli chickens under a Bayesian framework, outperforming alternative nonlinear models in predictive accuracy. Growth dynamics were strongly influenced by sex, with males achieving substantially higher asymptotic weights than females across all production systems, while also exhibiting slower growth rates and later inflection ages. In addition, the production system significantly modified growth trajectories, with the conventional floor system supporting the highest growth potential, particularly in males.

These findings indicated that growth differences were not limited to final body weight but also involved coordinated changes in growth rate and timing. The combined behavior of parameters, especially B and k, revealed that temporal shifts in growth trajectories played a key role in shaping overall development. The Bayesian nonlinear mixed modeling approach used in this study proved effective in capturing these multidimensional growth patterns and provided a robust analytical framework for future growth, breeding, and management studies. From a practical perspective, these findings suggested that growth modelling approaches integrating biological variability and environmental effects could provide valuable support for optimizing housing strategies, growth management, and breeding decisions in indigenous and dual-purpose poultry production systems. In particular, the superior growth performance observed under conventional floor conditions may have offered useful guidance for small-scale producers aiming to improve meat production efficiency in Denizli chickens.

## Figures and Tables

**Table 1:** Bayesian comparison of candidate nonlinear growth models using predictive accuracy and information criteria.

Model	LOOIC	ΔLOOIC	WAIC	Bayesian R^2^	RMSE	MAE	Rhat Max	Divergences
Gompertz	225.16	0.00	224.82	0.9997	13.06	8.95	1.002	0
Richards	236.07	10.91	234.74	0.9996	15.36	9.94	1.002	0
von Bertalanffy	241.69	16.52	241.52	0.9994	18.25	16.54	1.001	0
MMF	258.51	33.35	257.14	0.9990	22.61	19.84	1.005	0
Weibull	260.40	35.24	260.26	0.9989	26.02	22.13	1.004	0
Logistic	291.34	66.18	291.00	0.9963	45.22	34.76	1.002	0
Negative Exp.	317.65	92.49	317.57	0.9890	78.69	70.45	1.002	0
Brody	322.30	97.14	322.23	0.9867	86.16	75.78	1.001	0

LOOIC = Leave-One-Out Information Criterion; WAIC = Widely Applicable Information Criterion; Bayesian R^2^ = Bayesian coefficient of determination; RMSE = Root Mean Square Error; MAE = Mean Absolute Error; Rhat = potential scale reduction factor indicating convergence (values close to 1.00 indicate good convergence); Divergences = number of divergent transitions during Hamiltonian Monte Carlo sampling.

**Table 2:** Posterior estimates of population-level Gompertz model parameters.

Parameter	Estimate	SE	95% CrI
log(A)	7.867	0.006	7.856–7.879
log(B)	1.239	0.012	1.216–1.263
log(k)	−1.954	0.013	−1.980–−1.930
σ	14.64	2.31	11.03–19.92

A = asymptotic body weight; k = growth rate parameter; B = integration constant controlling curve shape; σ = residual standard deviation. CrI = credible interval derived from posterior distributions.

**Table 3:** Group-specific posterior estimates of Gompertz growth parameters.

System	Sex	A (g)	k	Inflection Age (Week)	Inflection Weight (g)
Cage	Female	2159 (2048–2278)	0.150 (0.146–0.153)	8.22 (8.03–8.41)	795 (754–838)
Cage	Male	2940 (2773–3108)	0.143 (0.139–0.146)	8.83 (8.62–9.05)	1082 (1020–1144)
Floor	Female	2241 (2087–2405)	0.145 (0.140–0.149)	8.47 (8.22–8.73)	825 (768–885)
Floor	Male	3322 (3077–3576)	0.129 (0.125–0.133)	9.75 (9.43–10.08)	1222 (1132–1316)
Enriched	Female	2173 (2031–2323)	0.148 (0.144–0.153)	8.33 (8.08–8.57)	799 (747–855)
Enriched	Male	3041 (2808–3289)	0.139 (0.135–0.144)	9.07 (8.76–9.38)	1119 (1033–1210)

A = asymptotic body weight; k = growth rate parameter; Inflection age = age at maximum growth rate; Inflection weight = body weight at inflection point. Values are presented as posterior mean with 95% credible intervals (CrI).

**Table 4:** Posterior contrasts between sexes within each production system.

System	Parameter	Difference (Male–Female)	95% CrI	Post. Prob.
Conventional cage	A (asymptotic weight)	780.2306	583.8988–979.9996	1.000
Conventional floor	A (asymptotic weight)	1080.4917	793.4191–1384.7130	1.000
Enriched floor	A (asymptotic weight)	868.1815	597.7634–1149.7210	1.000
Conventional cage	B (integration constant)	0.1081	0.0807–0.1363	1.000
Conventional floor	B (integration constant)	0.1077	0.0804–0.1359	1.000
Enriched floor	B (integration constant)	0.1084	0.0808–0.1367	1.000
Conventional cage	k (growth rate)	−0.0068	−0.0118–−0.0017	0.004
Conventional floor	k (growth rate)	−0.0158	−0.0219–−0.0097	0.000
Enriched floor	k (growth rate)	−0.0086	−0.0150–−0.0022	0.005
Conventional cage	Inflection age	0.6092	0.3166–0.8990	1.000
Conventional floor	Inflection age	1.2831	0.8763–1.6901	1.000
Enriched floor	Inflection age	0.7382	0.3494–1.1295	1.000
Conventional cage	Inflection weight	287.0308	214.8044–360.5217	1.000
Conventional floor	Inflection weight	397.4907	291.8826–509.4074	1.000
Enriched floor	Inflection weight	319.3861	219.9049–422.9587	1.000

Values represent posterior mean differences between males and females within each production system. Positive values indicate higher estimates in males, whereas negative values indicate higher estimates in females. CrI = credible interval. Post. Prob. = posterior probability that the male–female difference is greater than zero.

## Data Availability

The data that support the findings of this study are available from the corresponding author upon reasonable request. The R version 4.6.0 scripts used for Bayesian model fitting and posterior analyses are available from the corresponding author upon reasonable request.
